# Periodontal Inflamed Surface Area Is Associated With Increased Gestational Blood Pressure and Uric Acid Levels Among Pregnant Women From Rural North China

**DOI:** 10.3389/fcvm.2022.830732

**Published:** 2022-03-01

**Authors:** Shaonan Hu, Feifan Yu, Hong Jiang, Wei Shang, Hui Miao, Simin Li, Jianjiang Zhao, Hui Xiao

**Affiliations:** ^1^Innovation Center Computer Assisted Surgery, Leipzig University, Leipzig, Germany; ^2^School of Engineering and Applied Science, University of Pennsylvania, Philadelphia, PA, United States; ^3^Department of Obstetrics and Gynecology, Sichuan Academy of Medical Science, Sichuan Provincial People's Hospital, Chengdu, China; ^4^Heping Hospital Affiliated to Changzhi Medical College, Changzhi, China; ^5^Stomatological Hospital, Southern Medical University, Guangzhou, China; ^6^Shenzhen Stomatological Hospital, Southern Medical University, Shenzhen, China

**Keywords:** periodontal disease, low birth weight, gestational hypertension, uric acid, periodontal inflamed surface area

## Abstract

**Background:**

Periodontal disease has been associated with gestational complications and both conditions have a high prevalence in rural populations from developing regions. A cross-sectional study was carried out to explore the relationship between periodontal inflamed surface area (PISA), blood pressure (BP), and, serum uric acid levels (UA) in a group of rural North Chinese pregnant women in the third trimester of pregnancy.

**Methods:**

Three hundred and thirty-five rural women aged 20–34 years, with normal body mass index (BMI) were examined in a cross-sectional study during their third trimester of gestation. Exclusion criteria were history of pregnancy complications, multiple pregnancy, smoking habits, diabetes, hypertension or any known infectious disease. Socio-demographic variables, including age and socioeconomic status (SES), systolic blood pressure (SBP) and diastolic blood pressure (DBP) readings, serum UA levels, and PISA values were recorded. A structural equation model was implemented with two constructed latent variables including “Dem” (comprising of age and SES category to represent unobserved demographic variables) and, “BP” (comprising of SBP and DBP to account for measurement error and lack of multiple BP readings). The model accounted for co-variance of BP and UA, and implemented simultaneous regressions for BP and UA as outcomes, upon Dem and PISA values as exogenous variables.

**Results:**

The median PISA score was 1,081.7 (IQR = 835.01), reflecting high levels of periodontal inflammation in the sample. SEM showed a significant association of PISA with BP (estimate = 0.011, 95% CI = 0.009–0.012 *p* < 0.001) and UA (estimate = 0.001, 95% CI = 0.001–0.001, *p* < 0.001).

**Conclusion:**

Higher PISA values were significantly associated with higher blood pressure and uric acid levels among rural pregnant women in a cross-sectional sample from a center in North China after accounting for a latent demographic construct derived from age and SES.

## Introduction

Hypertensive disorders of pregnancy affect more than 5% of women in China, with the highest incidence in North China (1). Hypertension without the development of significant proteinuria (<0.3 g/l), after 20 weeks of gestation or during labor and/or within 48 h of delivery is termed gestational hypertension and is a risk for preeclampsia, eclampsia, and preterm low birth weight (2). Inflammation has been implicated in the pathogenesis of preeclampsia and attributed to chronic subclinical infections and higher levels of pro-inflammatory cytokines (3). Principally, gestational blood pressure and increased oxidative stress are interlinked (4) and both are associated with a higher risk of adverse pregnancy outcomes (5). Chronic infections are established contributors to these, operating through increased systemic pro-inflammatory cytokine load (6). Serum uric acid, a primary physiological antioxidant is considered as one of the primary markers of oxidative stress (7) and hyperuricemia in pregnancy is implicated both as a risk marker and contributory factor for preeclampsia (8). In particular, increased blood pressure during the third trimester of pregnancy is linked to adverse birth outcomes (9).

Periodontal infection is a source of chronic systemic microbial load and has been associated with the incidence of preeclampsia (10) and pre-term low birth weight infants (11). Proposed mechanisms of the periodontal infection-pregnancy outcome link include oral bacterial translocation to the uteroplacental unit, increased systemic oxidative stress and pro-inflammatory cytokine load leading to preeclampsia and premature rupture of membranes (12). A major contributory mechanism is the systematic dissemination and placental localization of periodontal pathogens such as *Porphyromonas gingivalis* with ensuing immuno-inflammatory sequelae (9, 13). Umbrella reviews of systematic reviews have noted that periodontal disease increases the odds of pre-eclampsia (14) and pre-term birth (15) by ~2-fold but also show that several meta-analyses have not adjusted for confounding. Heterogeneity in the strength association depends on the nature of exposure and outcome variables analyzed (16, 17). At the same time, current evidence evaluating the efficacy of periodontal treatment in reducing adverse pregnancy outcomes is unclear and incongruent (18–22). An umbrella review found significant effects upon subgroup analysis based on sociodemographic conditions (23), which highlights the significance of studying vulnerable populations in this context.

Periodontal disease can be highly prevalent among pre-conception women in some regions of China (24), compounded by low levels of oral health awareness and utilization of dental services in rural areas (25). Periodontal disease has been reported to have a high prevalence among rural pregnant women in China (26). Therefore, its potential impact on pregnancy-related variables and outcomes in rural Chinese populations needs greater investigation. In addition, a large-scale retrospective study showed an increased risk of hypertension was associated with periodontal disease in the Chinese population (27), further highlighting the necessity of investigating periodontal disease as an exposure in the context of gestational blood pressure in Chinese populations. Higher prevalence of both periodontitis (24, 25) and hypertensive disorders during pregnancy (1) in populations in underserved regions of North China begets further investigations into their association.

Traditional measures of periodontal disease and disease categories pose limitations in assessing the burden of active inflammation which is responsible for its systemic sequelae, and therefore, the Periodontal Inflamed Surfaced Area (PISA) index was developed (28), representing the net probing depth of bleeding on probing positive sites for an individual. PISA values comprise a continuous quantitative variable that prevents the loss of information inherent to the categorization of periodontal disease categories (29). Moreover, different case-definition criteria applied for periodontal diagnosis in pregnant women yield widely variable disease estimates, and PISA may be a more suitable measure of inflammatory burden (30). Furthermore, periodontal inflammation as measured by PISA values has been associated with high blood pressure in a large-scale study of over 8,000 subjects (31).

The present cross-sectional study aimed to explore the association of periodontal inflammatory burden assessed using PISA values with prenatal gestational blood pressure and serum uric acid levels in a sample of rural-living North Chinese women in the third trimester of pregnancy.

## Materials and Methods

Ethical approval and sample size estimation: The study protocol was approved by the Medical Ethics Committee of Changzhi Medical College. All study procedures were compliant with the Declaration of Helsinki. Sample size estimation was performed using the “epi.sssimpleestb” function from the epidemilogical R packge “EpiR” (https://cran.r-project.org/web/packages/epiR/index.html). Data for the estimated prevalence of gestational hypertension in North China (7.44%) (1) was used to estimate the sample size, assuming a population of 1,000 suitable pregnant women would visit the sampling center during the data collection period. At a confidence interval of 95% and margin of error 20%, the target sample size was determined as 352. However, 335 subjects finally participated in the study as 17 recruited subjects were unable or unwilling to complete the study procedures.

### Study Participants

A group of 335 eligible subjects in week 28 or beyond of pregnancy were recruited consecutively during their routine prenatal visit at The Heping Affiliated Hospital of Changzhi Medical College, Changzhi City, Shanxi Province, China, from January to April 2021. The inclusion criteria were; pregnant women aged between 18 and 34 years in the third trimester, Body Mass Index (BMI) of 18.5–24.9, presence of at least 20 teeth, with no history of periodontal or dental treatment in the past 1 year. Exclusion criteria were smoking or smoking habits, previous history of preterm delivery, previously diagnosed gestational hypertensive disorders, or other pregnancy complications, known systemic conditions or diseases such as chronic hypertension, diabetes, renal disease, polyhydramnios or known congenital malformation in fetus, infections during pregnancy such as bacterial vaginosis and chorioamnionitis, other bacterial or viral illnesses, or antibiotic use during pregnancy. All subjects provided written and oral informed consent before any study procedure. Oral health counseling was provided to all screened subjects by a dental professional irrespective of their study participation. Appropriate referral for dental or medical management was also provided when deemed necessary based on the clinical findings.

### Data Collection

Medical records were the source of demographic variables; age, socioeconomic level (SES), scored as high, medium or low, where to assess SES, monthly family income was noted and grouped into; low (<2,000 RMB per month), moderate (2,000–6,000 RMB per month) or High (6,000 RMB per month) (32), and retrospective medical data. The mean of three consecutive readings of blood pressure (SBP and DBP) was calculated and serum uric acid level (UA) was measured. A full mouth periodontal examination was performed by a trained single examiner (W.S) and included the recording of pocket probing depth (PPD), clinical attachment loss (CAL) and bleeding on probing (BOP) at 6 sites per tooth, by using a UNC-15 periodontal probe. PPD and CAL were measured as the linear distance in millimeters and rounded to the nearest millimeter, where PPD was the distance from the gingival margin to the base of the pocket and CAL was measured as the linear distance from the cementoenamel junction to the base of the pocket. Bleeding on probing (BOP) was recorded as a dichotomous variable representing presence of absence of bleeding. PISA values were calculated for each subject as described earlier (28) using an excel spreadsheet. In brief, the mean CAL and gingival recession for each tooth were recorded and were used to determine the periodontal epithelial surface area (PESA) score per tooth, representing the surface area in mm2 enveloped by pocket epithelium. Next, the PESA for score multiplied by the number of BOP positive sites around that tooth indicated the PISA score for that tooth, and the sum of these determined the full-mouth PISA score.

### Data Analysis

All data analysis was performed in the R statistical environment. Prior to modeling, variable distribution was examined for normality using the Shapiro Wilk test. Structural equation modeling (SEM) was applied using the “lavaan” package (v 0.5-9) (33) using a maximum likelihood estimator. SEM is a multivariate modeling technique that allows simultaneous modeling of observed and unobserved or latent variables, and these variables can account for measurement error and observed covariation (34, 35). In the SEM model, UA, SBP, DBP were utilized as observed endogenous or dependent variables, and, age, SES and PISA were modeled as observed exogenous or independent variables. Two latent variables were created, including, “BP” as a combination of endogenous variables SBP and DBP, and “Dem” as a combination of exogenous variables age and SES. The co-variance of BP with UA was modeled to account for residual correlation. Two simultaneous regressions were modeled for BP and UA, using Dem and PISA values as predictors. Model fit was estimated as acceptable where Tucker-Lewis Index (TLI) > 0.90 (36) and normed Chi-square value <5 (37).

## Results

**Figure 1:**
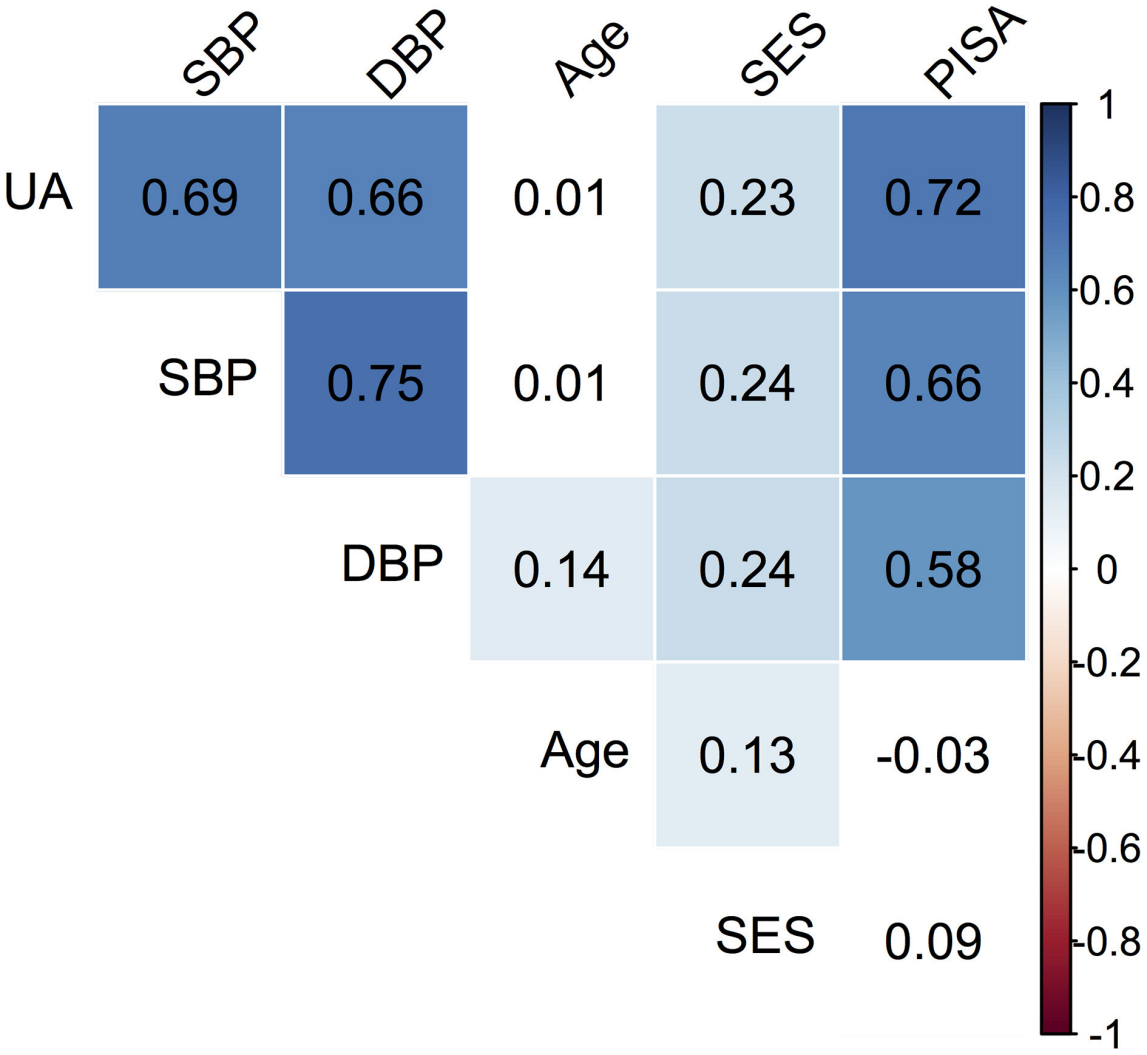
Correlation matrix plot of the observed variables. The colored boxes represent significant *p*-values (*p* < 0.05), where the listed value corresponds to the correlation coefficient. White boxes represent non-significant correlations. For SES, the reference category is Medium (M).

Descriptive data pertaining to the cross-sectional sample are presented in Table 1. The median age of the cohort was 24 (IQR = 6) years. The distribution of SES showed that 119 (35.5%) were in low SES and 216 (64.5%) were in the medium SES category. The mean PISA score was 1,081.7 (IQR = 835.01). The median SBP was 120 (IQR = 5) and DBP was 70 (IQR = 10), while median UA was 3.7 mg/dl (IQR = 0.6). Based on SBP and DBP reading cut-offs for gestational hypertension (1, 2), 11.3% women satisfied the criteria and were referred for additional investigations. However, it was recognized that single day BP readings were not sufficient for definitive diagnosis; hence this grouping was not utilized for data analysis. Significant correlations between the observed variables are summarized in a correlation matrix plot presented in Figure 1. Significant and strong correlations (*r* > 0.6, *p* < 0.001, all) were noted between the three endogenous dependent variables DBP, SBP, and UA. Among the exogenous variables, age was significantly but weakly correlated with DBP (*r* = 0.14, *p* = 0.01), lower SES was weakly correlated with SBP (*r* = 0.24, *p* < 0.001), DBP (*r* = 0.24, *p* < 0.001) and UA (*r* = 0.23, *p* < 0.001). PISA score was moderately correlated with DBP (*r* = 0.58, *p* < 0.001) and strongly correlated with SBP (*r* = 0.66, *p* < 0.001) and UA (*r* = 0.72, *p* < 0.001). Very weak but significant correlations were noted for lower SES with Age (*r* = 0.13, *p* = 0.02) and PISA score (*r* = 0.12, *p* = 0.03). The SEM model outcomes are summarized in Table 2. The latent variable BP was highly significantly predicted by PISA (estimate = 0.011, 95% CI = 0.009–0.012, *p* < 0.001) and also significantly predicted by Dem (estimate = 3.37, 95% CI = 0.41–6.83, *p* = 0.03). UA was also significantly predicted by PISA (estimate = 0.001, 95% CI = 0.001–0.001, *p* < 0.001) and also significantly predicted by Dem (estimate = 0.15, 95%ci = 0.13–0.29, *p* = 0.04).

**Table 1:** Descriptive statistics.

**Parameter**	**Range**	**Median (interquartile range)**
Age (years)	18–34	24 (6)
PISA value (mm^2^)	0.0–2,472.6	1,081.7(835.01)
SBP (mm of Hg)	110–160	120 (5)
DBP (mm of Hg)	70–100	70 (10)
UA (mg/dl)	3.1–5.0	3.7 (0.6)
	**Frequency**	
SES	Low = 119 (35.5%), Medium = 216 (64.5%)	

*PISA, Periodontal Inflamed Surface Area; SBP, Systolic Blood Pressure; DBP, Diastolic Blood Pressure; UA, Serum Uric Acid; SES, Socioeconomic status*.

**Table 2:** Structural relationships and variances in the SEM^a^ analysis.

**Structural relationship**	**Estimate**	**95% CI**	***P-*value**
PISA ➜ BP^b^	0.011	0.009–0.012	<0.0001^***^
Dem^c^ ➜ BP^b^	3.59	0.44–6.73	0.03^*^
PISA ➜ UA	0.001	0.001–0.001	<0.0001^***^
Dem^c^ ➜ UA	0.15	0.13–0.28	0.04^*^
**Variances**			
SBP	18.45	12.78–24.13	<0.0001^***^
DBP	15.48	11.97–18.99	<0.0001^***^
UA	0.09	0.07–0.12	<0.0001^***^
Age	14.45	12.12–16.78	<0.0001^***^
SES^*^	0.14	0.05–0.24	0.003
BP	24.56	12.50–36.62	<0.0001^***^
Dem	0.70	−0.34–1.74	0.19

*** *p < 0.0001*,

* *p < 0.05*.

a *Model fit indices: TLI = 0.96, normed Chi-square = 3.40*.

b *“BP” indicates a latent BP variable constructed from SBP and DBP*.

c *“Dem” indicates a latent Demographic variable constructed from Age and SES.*

*PISA, Periodontal Inflamed Surface Area; SBP, Systolic Blood Pressure; DBP, Diastolic Blood Pressure; UA, Serum Uric Acid; SES, Socioeconomic status*.

## Discussion

This cross-sectional study explored the association of gestational blood pressure and UA, a marker of oxidative stress with periodontal inflammatory burden among pregnant women at a rural center in North China, using structural equation modeling. While the present study, identified the nature of the association between periodontal inflammation, BP and a marker of oxidative stress among rural women using a cross-sectional design, epidemiological studies have linked periodontal disease with a higher risk of pregnancy complications (10, 11, 14, 15, 17, 19) including preeclampsia and preterm low birth weight deliveries (38, 39). The type of periodontal disease case-definition may influence the outcome of an association of periodontal disease with pregnancy complications as noted by Papapanou and Ide (40), who also found that case-control and cross-sectional studies provided a higher estimate of the association as compared to prospective studies. Therefore, a strength of the current study is the utilization of PISA as an exposure variable indicating the inflammatory burden arising from periodontal disease. The median PISA score was 1,081.7 (IQR = 835.01), reflecting high levels of active periodontal inflammation in this sample, largely in agreement with previous findings of a very high prevalence of periodontal disease among rural Chinese pregnant women (41). Of note, A PISA value ≥ 130.33 mm^2^ has been found to indicate periodontitis at very high sensitivity and specificity (42). Another strength of the present study is the use of SEM, a multivariate technique incorporating observed variables and latent constructs that allows for simultaneous modeling of multiple relationships and accounts for covariance between variables. Here, BP was modeled as a latent variable, as a trait derived from SBP and DBP (43), which can account for measurement errors inherent to the lack of longitudinal data, circadian pattern, and their correlation. Differences in circadian amplitudes of BP have been found to predict gestational complication even when BP readings were within physiological limits (44). In addition, 24-h BP during the third trimester has shown superior prediction of pregnancy complications than in-office BP readings, plausibly owing to the phenomenon of white coat hypertension (45). The restricted inclusion criteria were chosen to avoid the confounding effects of obesity, advanced age and other known systemic conditions. Age and income are established co-factors in the multi-factorial nature of gestational complications (46, 47) and were recorded. However, a number of confounding variables including the quality and regularity of prenatal care and diet that can influence gestational health. Considering these and other such variables were not assessed directly, a latent variable “dem” was constructed using maternal age and SES, as both of these factors are contributory to antenatal care utilization patterns, dietary variables, and pregnancy outcomes (48–50).

In the model, after accounting for the effects of latent demographic variables, higher gestational blood pressure was significantly associated with higher PISA values, in agreement with the results of Pietropaoli et al. (31) in the general population. Serum UA was also significantly associated with PISA score. Chronic infection and cytokine load have both been associated with increased xanthine oxidase activity (51) which is an important mediator of endogenous UA production. UA being a major plasma antioxidant and marker of systemic oxidative stress has been investigated with regards to periodontal disease and better periodontal status has been associated with higher levels of serum uric acid in healthy subjects (52). UA in pregnancy is considered an important risk marker of fetal risk in women with gestational hypertension (53) and may have a causal role in its etiology (54). These findings suggest a contributory role of periodontal inflammatory burden to systemic oxidative stress and hypertension occurring during pregnancy.

The major limitations of the current study include the cross-sectional design that precludes causal inferences, the small sample size, and possible effects of unaddressed predictors such as nutritional status, lifestyle-related risk factors, genotype, the possibility of occult infection, level of antenatal care level, physical activity, weight gain, sleep, psychological status, among others (16, 55–60). To compensate in part, a SEM model was thus designed to construct and model unobserved or latent dimensions and has been previously applied in periodontal research (61–65). Furthermore, this study design fails to address the possible syndemic nature of the association between periodontal inflammation and systemic state, whereby vulnerable subsets may be affected by both periodontal and other inflammatory conditions (66). Importantly, as this cross-sectional sample consisted of pregnant women classified previously as medically healthy and within a narrow age range and excluded advanced maternal age which is an independent risk factor for gestational hypertension (67), broader conclusions regarding the general populace in this region are precluded. As all subjects were recruited from those seeking care at a single tertiary center located in rural North China, selection bias may be applicable, as those reporting to small primary health care centers or those not seeking any antenatal care were not included. Thus, the present findings may be considered as the basis for further research using more robust study designs. In particular, future interventional studies investigating the effects of periodontal therapy on BP and UA are warranted in light of the present findings. Future studies should focus on shared risk factors (55–60), specific vulnerable subgroups (23) and optimal timing of intervention (68). A previous study has noted those receiving prophylactic treatment had a 7.6% incidence of low birth weight as compared to 10.1% among those not receiving any treatment (18). Considering that unexplained perinatal mortality has been linked with periodontal disease (69) and has a high prevalence in developing regions with low oral health awareness and accessibility, this preliminary data highlights that further research is essential to estimate the risk burden attributable to periodontal inflammation in mediating pregnancy complications in specific demographics. Such data would enable tailored public health measures to direct resource allocation within specific high-risk groups.

## Conclusion

A cross-sectional study using an SEM modeling approach showed that higher periodontal inflamed surface area was associated with increased gestational blood pressure and serum uric acid in a sample of rural North Chinese pregnant women. The quantum of risk imposed by periodontal inflammation and its clinical significance in this demographic warrants investigation in more appropriately designed large-scale, longitudinal studies.

## Data Availability Statement

The original contributions presented in the study are included in the article/supplementary material, further inquiries can be directed to the corresponding authors.

## Ethics Statement

The studies involving human participants were reviewed and approved by the Medical Ethics Committee of Changzhi Medical College, City of Changzhi, Shanxi Province, China (No. RT2021001). The patients/participants provided their written informed consent to participate in this study.

## Author Contributions

SH conceptualized the research idea, carried out data analysis, and led the paper writing. FY and HJ performed the data analysis. WS and HM were responsible for study design and data collection. SL, JZ, and HX initiated the idea of the study, supervised the project, and edited the manuscript. All authors contributed to the article and approved the submitted version.

## Funding

We appreciate the funding by the Science Research Cultivation Program of Stomatological Hospital, Southern Medical University (No. PY2020004), which was provided to support the postdoc research of Dr.rer.med., SL ( simin.li.dentist@gmail.com).

## Conflict of Interest

The authors declare that the research was conducted in the absence of any commercial or financial relationships that could be construed as a potential conflict of interest.

## Publisher's Note

All claims expressed in this article are solely those of the authors and do not necessarily represent those of their affiliated organizations, or those of the publisher, the editors and the reviewers. Any product that may be evaluated in this article, or claim that may be made by its manufacturer, is not guaranteed or endorsed by the publisher.

## References

[1] Ye C Ruan Y Zou L Li G Li C Chen Y. The 2011 Survey on hypertensive disorders of pregnancy (HDP) in China: prevalence, risk factors, complications, pregnancy and perinatal outcomes. PLoS ONE. (2014) 9:e100180. 10.1371/journal.pone.0100180 24937406 PMC4061123

[2] AbouZahr C Guidotti R. Hypertensive disorders of pregnancy. In: Murray CJL, Lopez AD, editors. Health Dimensions of Sex and Reproduction: The Global Burden of Sexually Transmitted Diseases, Maternal Conditions, Perinatal Disorders, and Congenital Anomalies. Geneva: WHO (1998), p. 219–41.

[3] López-Jaramillo P Herrera JA Arenas-Mantilla M Jáuregui IE Mendoza MA. Subclinical infection as a cause of inflammation in preeclampsia. Am J Ther. (2008) 15:373–6. 10.1097/MJT.0b013e318164c149 18645342

[4] Horton AL Boggess KA Moss KL Beck J Offenbacher S. Periodontal disease, oxidative stress, and risk for preeclampsia. J Periodontol. (2010) 81:199–204. 10.1902/jop.2009.090437 20151797 PMC4380012

[5] Schoots MH Gordijn SJ Scherjon SA van Goor H Hillebrands J-L. Oxidative stress in placental pathology. Placenta. (2018) 69:153–61. 10.1016/j.placenta.2018.03.003 29622278

[6] López-Jaramillo P Casas JP Serrano N. Preeclampsia: from epidemiological observations to molecular mechanisms. Braz J Med Biol Res. (2001) 34:1227–35. 10.1590/S0100-879X2001001000001 11593296

[7] Glantzounis GK Tsimoyiannis EC Kappas AM Galaris DA. Uric acid and oxidative stress. Curr Pharm Des. (2005) 11:4145–51. 10.2174/138161205774913255 16375736

[8] Bainbridge SA Roberts JM. Uric acid as a pathogenic factor in preeclampsia. Placenta. (2008) 29(Suppl. A):S67–72. 10.1016/j.placenta.2007.11.001 18093648 PMC3319018

[9] Bakker R Steegers EAP Hofman A Jaddoe VWV. Blood pressure in different gestational trimesters, fetal growth, and the risk of adverse birth outcomes. Am J Epidemiol. (2011) 174:797–806. 10.1093/aje/kwr151 21859836

[10] Kunnen A Van Doormaal JJ Abbas F Aarnoudse JG Van Pampus MG Faas MM. Review article: periodontal disease and pre-eclampsia: a systematic review. J Clin Periodontol. (2010) 37:1075–87. 10.1111/j.1600-051X.2010.01636.x 21070324

[11] Corbella S Taschieri S Del FM Francetti L Weinstein R Ferrazzi E. Adverse pregnancy outcomes and periodontitis: a systematic review and meta-analysis exploring potential association. Quintessence Int. (2016) 47:193–204. 10.3290/j.qi.a34980 26504910

[12] Ren H Du M. Role of maternal periodontitis in preterm birth. Front Immunol. (2017) 8:e00139. 10.3389/fimmu.2017.00139 28243243 PMC5303728

[13] Katz J Chegini N Shiverick KT Lamont RJ. Localization of *P. gingivalis* in preterm delivery placenta. J Dent Res. (2009) 88:575–8. 10.1177/0022034509338032 19587165 PMC3144059

[14] Daalderop LA Wieland BV Tomsin K Reyes L Kramer BW Vanterpool SF. Periodontal disease and pregnancy outcomes: overview of systematic reviews. JDR Clin. Transl. Res. (2018) 3:10–27. 10.1177/2380084417731097 30370334 PMC6191679

[15] Manrique-Corredor EJ Orozco-Beltran D Lopez-Pineda A Quesada JA Gil-Guillen VF Carratala-Munuera C. Maternal periodontitis and preterm birth: systematic review and meta-analysis. Commun Dent Oral Epidemiol. (2019) 47:243–51. 10.1111/cdoe.12450 30812054

[16] Vivares-Builes AM Rangel-Rincón LJ Botero JE Agudelo-Suarez AA. Gaps in knowledge about the association between maternal periodontitis and adverse obstetric outcomes: an umbrella review. J Evid Based Dent Pract. (2018) 18:1–27. 10.1016/j.jebdp.2017.07.006 29478679

[17] Pockpa ZAD Assem S Koffi-Coulibaly N Alexandre L Badran Z Struillou X. Periodontal diseases and adverse pregnancy outcomes: review of two decades of clinical research. Oral Health Prev Dent. (2021) 19:77–83. 10.3290/j.ohpd.b898969 33491381 PMC11641288

[18] Albert DA Begg MD Andrews HF Williams SZ Ward A Conicella ML. An examination of periodontal treatment, dental care, and pregnancy outcomes in an insured population in the United States. Am J Public Health. (2011) 101:151–6. 10.2105/AJPH.2009.185884 21088265 PMC3000729

[19] Bi WG Emami E Luo ZC Santamaria C Wei SQ. Effect of periodontal treatment in pregnancy on perinatal outcomes: a systematic review and meta-analysis. J Matern-Fetal Neonatal Med. (2021) 34:3259–68. 10.1080/14767058.2019.1678142 31630597

[20] Merchant AT Liu J Reynolds MA Beck JD Zhang J. Quantile regression to estimate the survivor average causal effect of periodontal treatment effects on birthweight and gestational age. J Periodontol. (2021) 92:975–82. 10.1002/JPER.20-0376 33155296

[21] Merchant AT Sutherland MW Liu J Pitiphat W Dasanayake A. Periodontal treatment among mothers with mild to moderate periodontal disease and preterm birth: reanalysis of OPT trial data accounting for selective survival. Int J Epidemiol. (2021) 47:1670–8. 10.1093/ije/dyy089 29868830

[22] Iheozor-Ejiofor Z Middleton P Esposito M Glenny AM. Treating periodontal disease for preventing adverse birth outcomes in pregnant women. Cochrane Database Syst Rev. (2017) 6:CD005297. 10.1002/14651858.CD005297.pub3 28605006 PMC6481493

[23] Rangel-Rincón LJ Vivares-Builes AM Botero JE Agudelo-Suarez AA. An umbrella review exploring the effect of periodontal treatment in pregnant women on the frequency of adverse obstetric outcomes. J Evid Based Dent Pract. (2018) 18:218–39. 10.1016/j.jebdp.2017.10.011 30077375

[24] Jiang H Su Y Xiong X Harville E Wu H Jiang Z Qian X. Prevalence and risk factors of periodontal disease among pre-conception Chinese women. Reprod Health. (2016) 13:141. 10.1186/s12978-016-0256-3 27903295 PMC5131524

[25] Qi X Qu X Wu B. Urban-rural disparities in dental services utilization among adults in China's megacities. Front Oral Health. (2021) 2:e673296. 10.3389/froh.2021.673296 35048016 PMC8757718

[26] Chen L Lu HX Wei TY Feng XP. Multiple factors analysis of periodontal status in pregnant women in Shanghai. Shanghai Kou Qiang Yi Xue. (2014) 23:452–6. 25338797

[27] Zhao MJ Qiao YX Wu L Huang Q Li BH Zeng XT. Periodontal disease is associated with increased risk of hypertension: a cross-sectional study. Front Physiol. (2019) 10:440. 10.3389/fphys.2019.00440 31105578 PMC6494953

[28] Nesse W Abbas F Van Der Ploeg I Spijkervet FK Dijkstra PU Vissink A. Periodontal inflamed surface area: quantifying inflammatory burden. J Clin Periodontol. (2008) 35:668–73. 10.1111/j.1600-051X.2008.01249.x 18564145

[29] Park SY Ahn S Lee JT Yun PY Lee YJ Lee JY. Periodontal inflamed surface area as a novel numerical variable describing periodontal conditions. J Periodontal Implant Sci. (2017) 47:328–38. 10.5051/jpis.2017.47.5.328 29093989 PMC5663669

[30] Conceição SD Gomes-Filho IS Coelho JM Brito SM Silva RB Batista JE. An accuracy study of the clinical diagnosis of periodontitis in pregnant women. J Periodontol. (2021) 92:1243–51. 10.1002/JPER.20-0441 33252149

[31] Pietropaoli D Del Pinto R Ferri C Marzo G Giannoni M Ortu E. Association between periodontal inflammation and hypertension using periodontal inflamed surface area and bleeding on probing. J Clin Periodontol. (2020) 47:160–72. 10.1111/jcpe.13216 31680283

[32] Qiu Y Huang Y Wang Y Ren L Jiang H Zhang L Dong C. The role of socioeconomic status, family resilience, and social support in predicting psychological resilience among chinese maintenance hemodialysis patients. Front Psychiatry. (2021) 12:723344. 10.3389/fpsyt.2021.723344 34658959 PMC8514615

[33] Rosseel Y. Lavaan: an R package for structural equation modeling and more. Version 0.5–12 (BETA). J Stat Soft. (2012) 48:1–36. 10.18637/jss.v048.i02

[34] MacCallum RC Austin JT. Applications of structural equation modeling in psychological research. Annu Rev Psychol. (2000) 51:201–26. 10.1146/annurev.psych.51.1.201 10751970

[35] Beran TN Violato C. Structural equation modeling in medical research: a primer. BMC Res notes. (2010) 3:1–0. 10.1186/1756-0500-3-267 20969789 PMC2987867

[36] Bentler PM Bonett DG. Significance tests and goodness of fit in the analysis of covariance structures. Psychol Bull. (1980) 88:588–606.

[37] Schumacker RE Lomax RG. A Beginner's Guide to Structural Equation Modeling. New York, NY: Psychology Press (2004).

[38] Offenbacher S Lieff S Boggess KA Murtha AP Madianos PN Champagne CM. Maternal periodontitis and prematurity. Part I: obstetric outcome of prematurity and growth restriction. Ann Periodontol. (2001) 6:164–74. 10.1902/annals.2001.6.1.164 11887460

[39] López NJ Smith PC Gutierrez J. Higher risk of preterm birth and low birth weight in women with periodontal disease. J Dent Res. (2002) 81:58–63. 10.1177/002203450208100113 11820369

[40] Ide M Papapanou PN. Epidemiology of association between maternal periodontal disease and adverse pregnancy outcomes–systematic review. J Clinical Periodontol. (2013) 40:S181–94. 10.1111/jcpe.12063 23627328

[41] Lu HX Xu W Wong MC Wei TY Feng XP. Impact of periodontal conditions on the quality of life of pregnant women: a cross-sectional study. Health Qual Life Outcomes. (2015) 13:1–4. 10.1186/s12955-015-0267-8 26018650 PMC4446953

[42] Leira Y Martín-Lancharro P Blanco J. Periodontal inflamed surface area and periodontal case definition classification. Acta Odontol Scand. (2018) 76:195–8. 10.1080/00016357.2017.1401659 29129119

[43] Song YE Morris NJ Stein CM. Structural equation modeling with latent variables for longitudinal blood pressure traits using general pedigrees. BMC Proc. (2016) 10:303–07. 10.1186/s12919-016-0047-4 27980653 PMC5133482

[44] Hermida RC Ayala DE Moj Fernez JR Alonso I Silva I. Blood pressure patterns in normal pregnancy, gestational hypertension, and preeclampsia. Hypertension. (2000) 36:149–58. 10.1161/01.HYP.36.2.149 10948070

[45] Bellomo G Narducci PL Rondoni F Pastorelli G Stangoni G Angeli G. Prognostic value of 24-hour blood pressure in pregnancy. JAMA. (1999) 282:1447–52. 10.1001/jama.282.15.1447 10535435

[46] Schempf AH Branum AM Lukacs SL Schoendorf KC. Maternal age and parity associated risks of preterm birth: differences by race/ethnicity. Paediatr Perinat Epidemiol. (2007) 21:34–43. 10.1111/j.1365-3016.2007.00785.x 17239177

[47] Silva LM Coolman M Steegers EA Jaddoe VW Moll HA Hofman A. Low socioeconomic status is a risk factor for preeclampsia: the Generation R Study. J Hypertens. (2008) 26:1200–8. 10.1097/HJH.0b013e3282fcc36e 18475158

[48] Kim MK Lee SM Bae SH Kim HJ Lim NG Yoon SJ. Socioeconomic status can affect pregnancy outcomes and complications, even with a universal healthcare system. Intl J Equity Health. (2018) 17:1–8. 10.1186/s12939-017-0715-7 29304810 PMC5756361

[49] Marvin-Dowle K Kilner K Burley V Soltani H. Differences in dietary pattern by maternal age in the Born in Bradford cohort: a comparative analysis. PLoS ONE. (2018) 13:e0208879. 10.1371/journal.pone.0208879 30543673 PMC6292636

[50] Sui Y Ahuru RR Huang K Anser MK Osabohien R. Household socioeconomic status and antenatal care utilization among women in the reproductive-age. Front Public Health. (2021) 9:724337. 10.3389/fpubh.2021.724337 34589464 PMC8475754

[51] Moriwaki Y Yamamoto T Higashino K. Enzymes involved in purine metabolism–a review of histochemical localization and functional implications. Histol Histopathol. (1999) 14:1321–40. 10506947 10.14670/HH-14.1321

[52] Brotto RS Vendramini RC Brunetti IL Marcantonio RA Ramos AP Pepato MT. Lack of Correlation between Periodontitis and Renal Dysfunction in Systemically Healthy Patients. Eur J Dent. (2011) 5:8–18. 21228952 PMC3019747

[53] Roberts JM Bodnar LM Lain KY Hubel CA Markovic N Ness RB. Uric acid is as important as proteinuria in identifying fetal risk in women with gestational hypertension. Hypertension. (2005) 46:1263–9. 10.1161/01.HYP.0000188703.27002.14 16246973

[54] Kang DH Finch J Nakagawa T Karumanchi SA Kanellis J Granger J. Uric acid, endothelial dysfunction and pre-eclampsia: searching for a pathogenetic link. J Hypertens. (2004) 22:229–35. 10.1097/00004872-200402000-00001 15076175

[55] Downs DS Chasan-Taber L Evenson KR Leiferman J Yeo S. Physical activity and pregnancy: past and present evidence and future recommendations. Res Q Exerc Sport. (2012) 83:485–502. 10.5641/027013612804582669 23367811 PMC3563105

[56] Blyton DM Skilton MR Edwards N Hennessy A Celermajer DS Sullivan CE. Treatment of sleep disordered breathing reverses low fetal activity levels in preeclampsia. Sleep. (2013) 36:15–21. 10.5665/sleep.2292 23288967 PMC3524539

[57] Cetin O Guzel Ozdemir P Kurdoglu Z Sahin HG. Investigation of maternal psychopathological symptoms, dream anxiety and insomnia in preeclampsia. J Matern-Fetal Neonatal Med. (2017) 30:2510–5. 10.1080/14767058.2016.1254185 27806675

[58] Fong FM Sahemey MK Hamedi G Eyitayo R Yates D Kuan V. Maternal genotype and severe preeclampsia: a HuGE review. Am J Epidemiol. (2014) 180:335–45. 10.1093/aje/kwu151 25028703

[59] Raju K Berens L. Periodontology and pregnancy: an overview of biomedical and epidemiological evidence. Periodontology 2000. (2021) 87:132–42. 10.1111/prd.12394 34463990

[60] Beckers KF Sones JL. Maternal microbiome and the hypertensive disorder of pregnancy, preeclampsia. Am J Physiol Heart Circ. (2020) 318:H1–0. 10.1152/ajpheart.00469.2019 31626558

[61] Fisher MA Taylor GW West BT McCarthy ET. Bidirectional relationship between chronic kidney and periodontal disease: a study using structural equation modeling. Kidney Int. (2011) 79:347–55. 10.1038/ki.2010.384 20927035 PMC3045269

[62] Nascimento GG Leite FR Peres KG Demarco FF Corrêa MB Peres MA. Metabolic syndrome and periodontitis: a structural equation modeling approach. J Periodontol. (2019) 90:655–62. 10.1002/JPER.18-0483 30447085

[63] Hwang SY Yang JY Kim KE. Relationship between socioeconomic status and periodontal disease using Structural Equation Modeling. J Korean Soc Dental Hyg. (2018) 18:979–86. 10.13065/jksdh.2018008433759000

[64] Machado V Botelho J Proença L Alves R Oliveira MJ Amaro L. Periodontal status, perceived stress, diabetes mellitus and oral hygiene care on quality of life: a structural equation modelling analysis. BMC Oral Health. (2020) 20:1–11. 10.1186/s12903-020-01219-y 32819351 PMC7441730

[65] Rawlinson A Vettore MV Baker SR Robinson PG. Periodontal treatment, psychological factors and oral health-related quality of life. J Clin Periodontol. (2021) 48:226–36. 10.1111/jcpe.13405 33263182

[66] Bartold PM Mariotti A. The future of periodontal-systemic associations: raising the standards. Curr Oral Health Rep. (2017) 4:258–62. 10.1007/s40496-017-0150-2 28944159 PMC5587612

[67] Kahveci B Melekoglu R Evruke IC Cetin C. The effect of advanced maternal age on perinatal outcomes in nulliparous singleton pregnancies. BMC Preg Child. (2018) 18:1–7. 10.1186/s12884-018-1984-x 30134873 PMC6106883

[68] Xiong X Buekens P Goldenberg RL Offenbacher S Qian X. Optimal timing of periodontal disease treatment for prevention of adverse pregnancy outcomes: before or during pregnancy? Am J Obstet Gynecol. (2011) 205:111–e1. 10.1016/j.ajog.2011.03.017 21620355

[69] Shub A Wong C Jennings B Swain JR Newnham JP. Maternal periodontal disease and perinatal mortality. Aust N Z J Obstet Gynaecol. (2009) 49:130–6. 10.1111/j.1479-828X.2009.00953.x 19441161

